# Ambidexterity

**Published:** 1891-02

**Authors:** 


### Ambidexterity.

There are perhaps few accomplishments so useful to a dentist as the power of employing at will the left as well as the right hand. And yet comparatively few persons acquire the facility of using the hands inter-changeably. It is a very curious circumstance that the neglected use of the left member seems to be the result of a widespread aversion to the practice of replacing it for its neighbor. We go a long way back in history when we attempt to discover a period when the hands were regarded as of equal value. The very words *right* and *left*, and their analogues in the dead and continental languages, imply something underhanded in connection with those who work with the left, but uprightness, probity, and straightforwardness when the right hand is employed. Dexterity suggests aptitude and skilled performance of some delicate manipulation, whilst the sinister action connotes it may be skill, but brought to bear in a way that is not for the benefit of the persons concerned. A left-handed blow is a blow in the dark, and many are the curious charms, invocations to evil spirits, which constitute a large portion of folklore in the less civilized villages in the United Kingdoms containing directions where the word "left" appears figuring in a truly sinister way. However, it would seem that prejudice enters in no small measure into the causation of the disrepute into which the left hand has fallen. The majority of individuals are content to employ half their faculties and half their manual possibilities. Custom, it would appear, has induced mankind to favor the "right" and neglect the "left," a neglect which has resulted in a less skill and adroitness in that hand: hence has arisen clumsiness, inaptitude, or even manipulative failure, and from these, of course, obloquy. How far nature has given us a bias towards adroitness, that is right-handed action, is a moot point, for children as a rule employ one or the other hand apparently as the fancy dictates, until taught by the minatory slap from the guardians of their tender years. Indeed, the frequency with which children evince ambidexterity encourages the belief that this faculty is almost inherent in the average individual. Many of our readers may remember how strongly the late Charles Reade, the novelist, insisted upon this. Even if the natural state of affairs is that the majority of mankind are born, grow up, and die rignt-handed, there can be very little doubt that careful training, more especially during youth, can accomplish almost perfect ambidexterity. The degree of success in any given case will, of course, depend upon the general handiness of the individual. The average man bungles at threading a needle, a feat which is accomplished without apparent effort by the most unneathanded of Phyllises. Here education and use come in, and it is the object of the present article to insist upon the value to dentists of educating the left hand to do what the right hand is capable of doing. The enormous power of substitution in nature can not but prove to us that we are dependent upon one set of members only so far as we neglect making use of auxiliaries. Handless monsters acquire a nicety of touch and facility of use with the toes, which carries them to the fore front of skilled workers with paint brush, pencil, etc. Persons afflicted with writers' cramp, and those who have, through accident, lost the use of their right hand, soon acquire the art of writing and drawing, and general manipulation with their left. The spur which necessity brings to bear in these cases would not be needed were a commonsense view of the matter taken by mankind at large, and the young of either sex duly instructed in the use of both hands. We do not for the moment wish to imply that ambidexterity may not be acquired in later life, and would urge our readers, as far as possible, to cultivate the left hand, unless they should be what is called "left handed," for then the right hand should receive its share of the useful, if irksome training. None but those who have tried it will be able to appreciate the usefulness of ambidexterity in dentistry. It enables difficult manipulations to be performed with increased facility, and forms the means of lessening the labors, and relieving the monotony of prolonged operations.—*Dental Record.*

